# Effects of Dietary Supplementation with Tea Residue on Growth Performance, Digestibility, and Diarrhea in Piglets

**DOI:** 10.3390/ani14040584

**Published:** 2024-02-09

**Authors:** Chunfeng Wang, Yan Zhong, Han Liu, Hanmin Wang, Yali Li, Qiye Wang, Jianzhong Li, Pengfei Huang, Huansheng Yang

**Affiliations:** Hunan Provincial Key Laboratory of Animal Intestinal Function and Regulation, College of Life Sciences, Hunan Normal University, Changsha 410081, China

**Keywords:** tea residue, weaned piglets, intestinal health, chloride channels, diarrhea, immune

## Abstract

**Simple Summary:**

Tea residue is a by-product of tea product consumption and has potential as a feed additive for livestock and poultry feeding. This paper investigated the effects of fermented Fuzhuan tea residue on nutrient digestibility, growth performance, diarrhea condition, intestinal morphology and structure, ion channel expression level, and biochemical and antioxidant indexes in the plasma of weaned piglets. It was confirmed that tea residue can improve diarrhea in weaned piglets, and the functional anti-diarrhea mechanism of tea was preliminarily revealed by whole-cell patch clamp detection, providing a reference for the rational utilization of tea residue in feeding production.

**Abstract:**

Thirty-six healthy 21-day-old weaned ternary piglets (Duroc × Landrace × Yorkshire) were randomly divided into two treatments with 18 replicates per treatment and one pig per replicate. The control group was fed with a basal diet and the test group was fed with diets supplemented with 1 kg/t tea residue. The test period was 28 days. The results are as follows: The addition of tea residue in the diet had no significant effect on the growth performance of weaned piglets (*p* > 0.05), but it could significantly reduce the diarrhea rate of piglets from 1 to 7 days and 1 to 28 days (*p* < 0.05). Compared with the control group, the dietary supplementation of tea residue had no significant effect on nutrient apparent digestibility, plasma biochemical indexes and plasma immune indexes (*p* > 0.05) but increased the content of glutathione in plasma (*p* < 0.05). Tea residue had no significant effect on the morphology of the jejunum and ileum of piglets (*p* > 0.05), but it could significantly reduce the content of chloride ions in feces (*p* < 0.05). Compared with the basal diet group, there was no significant difference in the relative expression of *TMEM16A* and *CFTR* mRNA in the colon of weaned piglets (*p* > 0.05). The whole-cell patch clamp recording showed that the *TMEM16A* and *CFTR* ion channels could be activated by ionomycin and forskolin, respectively. However, when HT-29 cells transfected with *TMEM16A* and *CFTR* channels were treated with tea residue extract, it could significantly inhibit the chloride current of the *TMEM16A* and *CFTR* ion channels (*p* < 0.05).

## 1. Introduction

Early weaning of piglets can make them prone to diarrhea. Weaning stress leads to insufficient secretion of digestive enzymes in the gastrointestinal tract of piglets and, coupled with changes in the environment of the digestive tract itself, leads to easy infection of pathogenic bacteria and viruses in the intestines of piglets, resulting in diarrhea in weaned piglets [1]. The post-weaning period is characterized by a high incidence of intestinal disorders in piglets with diarrhea and growth performance inhibition [2]. Post-weaning diarrhea is a disease that causes great economic losses [3]. A study found that post-weaning piglet mortality due to intestinal health problems ranges from 6% to 10% and sometimes up to 20% [4]. The imbalance of intestinal ion absorption and secretion is an important pathological mechanism of diarrhea, and many forms of diarrheal diseases depend on a related imbalance between the secretory and absorptive functions of the intestinal epithelium [5]. Diarrhea can be caused by increased secretion of osmotically active electrolytes (secretory diarrhea) or increased intake of osmotically active substances (osmotic diarrhea) [6]. When the osmotic pressure in the intestinal lumen is higher than in the intestinal wall, a large amount of water from the intestinal wall seeps into the intestinal lumen, causing the intestinal canal to dilate and peristalsis to accelerate, producing diarrhea [6], where the concentration of Na^+^ and K^+^ is the basis of osmotic diarrhea. Intestinal mucosal injury, such as inflammation and ulceration, leads to increased local vascular permeability and leakage of proteins and glucose into the intestine, resulting in increased osmotic pressure in the digestive tract and triggering diarrhea [7]. Diarrhea occurs when electrolytes with osmotic activity (e.g., Cl^−^) are actively secreted into the intestinal lumen for various pathological reasons, leading to a passive influx of water into the intestinal lumen, increasing the volume of the intestinal lumen contents and accelerating intestinal peristalsis [8]. Therefore, the regulation of intestinal ion transport in piglets may have an ameliorating effect on post-weaning diarrhea.

A large amount of tea residue is produced during the deep processing of tea. It has been reported that about 90% of tea leaves become tea residue after processing and consumption [9]. With Chinese companies producing tea drinks alone leaving about 180,000 to 200,000 tons of tea residue annually, India produces about 190,000 tons of tea residue every year. The production of tea residue brings a heavy economic burden. If it is not fully utilized, it will not only waste a lot of biological resources but also pollute the ecological environment. In addition, various active ingredients in tea residue can be used as an important source of medicinal products. Multiple beneficial components in tea residue (such as protein, tea polyphenols, polysaccharides, vitamins and minerals) promote animal health. Different bioactive components have a role in regulating various physiological and metabolic disorders [10]. Tea residue is rich in proteins, up to 21–28 g/100 g dry weight, and has functional activities such as antioxidant, antimutagenic and hypolipidemic [11] and, therefore, a high nutritional value. Studies have shown that green tea protein hydrolysates show antihypertensive activity [12]. Tea polyphenols have received attention from researchers and the public for their potential health functions, such as anticancer [13], antioxidant [14], hypoglycemic [15], anti-cardiovascular disease and antibacterial activities. Studies have shown that tea polyphenols can inhibit neuroinflammation and prevent degenerative effects in the brain and can be considered potential neuroprotective substances [16]. It has been shown that the source and composition of fibrous carbohydrates in calf rations can influence the digestion and utilization of nutrients by calves [17]. When tea dietary fiber was prepared from tea residues and fed to mice on a high-fat diet, it was found to reduce total serum and liver cholesterol and decrease lipid peroxidation [18]. There is much evidence that herbs such as tea can be effective against diarrhea [19]. Studies suggest that green tea may be a possible complementary treatment for acute radiological diarrhea [20], and the intake of a certain amount of green tea extract can improve diarrhea [21]. Fermented tea has been used for the treatment of pediatric diarrhea in the Hunan and Sichuan regions of China since long ago, but the mechanism is not well understood. Tea residue resources are abundant in China, and tea residue components are beneficial to health, are green and healthy, have non-toxic side effects, and meet the needs of pig industry feed additives. If tea residue can be used in the pig industry, it can not only solve the management problem of tea waste, ensuring the sustainable development and utilization of tea residue, but also greatly improve the added value of tea residue.

In this paper, by analyzing the effects of fermented Fuzhuan tea residue on growth performance, nutrient digestibility, diarrhea condition, intestinal morphology and structure, ion channel expression level, and biochemical and antioxidant indexes in the plasma of weaned piglets, the effects of tea residue on weaned piglets were explored, and the functional anti-diarrhea mechanism of tea was preliminarily revealed by whole-cell patch clamp detection.

## 2. Materials and Methods

### 2.1. Experimental Materials

#### 2.1.1. Tea Residue

The tea residue was derived from “Jinxiangyi” Fuzhuan tea produced by Yiyang Tea Factory in Hunan Province. The tea leaves were soaked in hot water at 80 °C for 5 min, the supernatant was discarded, the tea residue was collected and dried, followed by processing in a crushing machine, and stored at −80 °C.

#### 2.1.2. Tea Residue Filtrate

A 250 mL three-necked flask was taken, and 10 g of tea residue powder was weighed and added to 100 mL of water, which was placed in a 37 °C water bath and stirred for 2 h. After cooling, the supernatant was filtered and taken for cell experiments.

#### 2.1.3. Cell Lines and Plasmids

Human colon adenocarcinoma HT-29 cells and pEGFP-N1 plasmids were isolated, cultured and constructed by our own laboratory.

### 2.2. Animal Ethics Statement

According to the plan approved by the Animal Committee of Hunan Normal University, animal tests were carried out on the farm of Liuan Agricultural Science and Technology Comprehensive Development Co., Ltd., Liuyang City, Hunan Province, China.

### 2.3. Animals and Experimental Design

Thirty-six 21-day-old healthy weaned piglets with similar initial body weights (Duroc × Landrace × Yorkshire) were randomly divided into 2 treatments with 18 replicates per treatment and 1 pig per replicate. The control group was fed with the basal diet, and the experimental group was fed 1 kg/t of tea residue added to the basic diet, and this dose was formulated according to the pre-experimental results. The pre-feeding period was 3 days and the formal feeding period was 28 days. During the trial period, the piglets were fed 5 times a day, and the piglets were allowed to feed and drink freely and were kept in a single pen. The base diet was configured in accordance with the nutritional levels of weaned piglets in NRC (2012) [22]. The composition and nutrient content of the diets are shown in Table 1.

### 2.4. Sample Collection and Measurement

The abnormal behavior and diarrhea of the piglets were recorded daily throughout the test cycle. The piglets’ feces were collected separately for 3 consecutive days on the 5th, 6th, 7th, 26th, 27th and 28th days of the experiment, and the collected feces were mixed and stored at −20 °C. On days 0, 7 and 28, the piglets were fasted (free to drink) after 10 pm, then weighed and recorded the next morning, and the average daily gain (ADG) was calculated. The amount of feed added and residuals per day were also recorded in detail to calculate the average daily feed intake (ADFI), and the feed utilization rate of piglets (G:F) was calculated according to the formula.

On days 1 and 21 of the experiment, 0.2% chromium trioxide was added to the diet to determine nutrient digestibility. On the 7th and 28th days of the experiment period, 6 piglets in each group were randomly selected for anterior vena cava blood collection, and the plasma was centrifuged and stored in the refrigerator at −80 °C. Feed samples of 1 kg were collected from each group separately and stored at −80 °C. At the end of the experiment, 6 piglets were randomly selected from each group. The piglets were euthanized by intravenous injection of 4% sodium pentobarbital solution. The intestine was isolated and the mesentery was removed. After rinsing with physiological saline, 2 cm molecular samples of the jejunum, ileum and colon were taken, wrapped in tin foil, frozen in liquid nitrogen and then stored in a −80 °C refrigerator. About 2 cm of jejunum and ileum were taken separately and immersed in 10% formaldehyde solution; the solution was changed after 24 h and stored at 4 °C.

### 2.5. Determination of Nutrient Digestibility

When the test was finished, the fecal samples were taken out and the 3-day fecal samples were mixed well. The samples were washed with 2% H_2_SO_4_ solution, baked in an oven (65 °C) for 72 h [23] and stored in a refrigerator at −80 °C. The crude protein, crude fiber, crude fat and total energy digestibility in the rations and manure samples were determined according to the methods in GB/T 6432-2018 [24], GB/T 6433-2006, GB/T 6434-2006 and Feed Analysis and Feed Quality Testing Techniques, respectively. The chromium (Cr) content was determined by an atomic absorption spectrometer (Tokyo, Japan) according to the national standard GB/T 13088-2006.

### 2.6. Plasma Biochemical Indicators

Plasma was removed from the refrigerator, thawed, centrifuged and analyzed for biochemical, antioxidant and immune indexes using Cobas C311 (Roche Diagnostics, Zurich, Switzerland). Indicators included glutathione aminotransferase (AST), total protein (TP), alanine aminotransferase (ALT), alkaline phosphatase (ALP), albumin (ALB), cholesterol (TC), globulin (GLO), triglycerides (TG), high-density lipoprotein (HDL), low-density lipoprotein (LDL), catalase (CAT), glutathione (GSH), glutathione peroxidase (GSH-Px), malondialdehyde (MDA), superoxide dismutase (T-SOD), immunoglobulins (IgG, IgA, IgM) and total antioxidant capacity (T-AOC). The above kits were purchased from Weifang Sanwei Biological Engineering Co., Ltd., Weifang, China.

### 2.7. Morphological Structure of the Intestine

Samples of fixed jejunum and ileal intestinal tissue were removed, embedded in 1.5 cm sections of the intestine and cut into 4 mm thick sections. The sections were dried and stained with hematoxylin–eosin. Afterward, intestinal morphology was observed and photographed with an orthochromatic fluorescence microscope (version 4.12, Leica Imaging Ltd., Cambridge, UK). Villi height (VH) and crypt depth (CD) were measured with Image Pro Plus 6.0 software, and the ratio of villi height to crypt depth (VH:CD) was calculated for each small intestine. At least 30 intact villous crypts were randomly selected and measured for each sample.

### 2.8. Determination of Mineral Fractions in Feces

The fecal samples were taken out from the refrigerator, and the contents of mineral components such as calcium, phosphorus, sodium and chloride were detected. A total of 0.20 g of the crushed samples was weighed and placed in a microwave digestion tank, 10 mL of concentrated nitric acid and 1 mL of hydrogen peroxide were added in turn, and the samples were digested using a microwave digestion apparatus. After boiling, another 0.5 mL of perchloric acid was added into the digestion tank and the acid was driven away for 2 h at 180 °C. Finally, the contents of calcium, phosphorus, sodium and chlorine in the samples were determined by an inductively coupled plasma emission spectrometer (Agilent 5110 ICP-OES) with 1% nitric acid at a constant volume of 10 mL. Their detection was conducted according to GB/T 19203-2003, GB/T 6437-2018 [24], GB/T 40461-2021 and GB/T 24890-2010, respectively.

### 2.9. RNA Extraction and cDNA Synthesis

Total RNA was isolated from jejunal and colonic molecular sample tissues using TRIZOL reagent (TaKaRa, Beijing, China). mRNA reverse transcription kit (Prime Script TMRT Master Mix, Takala, Japan) instructions were followed: genomic DNA was removed first, followed by reverse transcription for cDNA synthesis.

### 2.10. Real-Time Fluorescence Quantitative PCR

Primer design was performed using Primer premier 5.0, primer sequences were verified for specificity using Primer-BLAST, and finally, primers were synthesized by Biotech. The primers and cDNA were diluted according to the recommended ratio in the reagent instructions. The reaction system (Quant-Studio, Thermo Fisher Scientific, Waltham, MA, USA) was added, then transferred to a 384-well plate, mixed and reacted in a real-time fluorescence quantitative PCR instrument (ABI 7900HT, USA). The relative expression of mRNA in the target gene was corrected with β-actin [25]. The primer sequences are shown in Table 2.

### 2.11. Target Gene Cloning and Plasmid Recombination

The enzymatic primers were designed using Snapgene 5.0.5 software, and the PCR reactions were performed using the primers with cDNA reported in Table 3. The sample was purified using a PCR purification kit (Beyoncé, D0033). The target gene *TMEM16A* and the pEGFP-N1 vector were cut by Bmt*I*/Bam*HI*enzyme, and the target gene *CFTR* and the pEGFP-N1 vector were cut by Xho*I*/Age*I*enzyme. Gel Extraction Kit D2500 (Omega) was used to recover enzymic PCR products and the pEGFP-N1 vector, which were then linked with T4 DNA ligase to transform *Escherichia coli* receptor cells DH5α (Thermo Fisher, EC0112) and select cloned plasmids. The correct plasmid was selected for DNA sequence analysis after enzyme digestion identification.

### 2.12. Transformation, Extraction and Purification of Recombinant Plasmids

The receptor cells were mixed with the recombinant plasmid, and the culture was expanded by thermal shock, then the culture was applied on LB agar (containing neomycin) plates and incubated in a constant-temperature chamber at 37 °C for 12–16 h. The monoclonal clones containing neomycin resistance were inoculated in LB liquid medium. A 2 mL sample of the DH5α bacterial solution identified as positive was sent to Shanghai Meiji Biomedical Technology Co., Shanghai, China.

### 2.13. Transient Transfection of Cells

HT-29 cells were cultured until the cell density was about 70% to 90% and washed twice with PBS, 1 mL of serum-free medium was added, then the diluted plasmids to be transfected and transfection reagent were added and mixed, and the culture was continued with normal culture medium after 4–6 h. The cells expressing EGFP were observed by inverted fluorescence microscopy for the preliminary detection of plasmid expression. Electrophysiological tests could be performed 24–48 h after transfection.

### 2.14. Electrophysiological Recordings

The whole-cell patch clamp experiment was performed at room temperature. The pEGFP-N1-CFTR and pEGFP-N1-TMEM16A HT-29 cells were taken separately and cultured on small slides, and after the cells were plastered, the cell smears were gently rinsed with Tait’s solution 2–3 times and placed in a perfusion bath. The perfusion speed was adjusted; a Ag/AgCl electrode was installed so that the electrode resistance in the bath was 3–5 MΩ; the inverted phase contrast microscope (Olympus, Tokyo, Japan) was adjusted, the micromanipulator was moved close to the cells to be examined, and the positive pressure inside the electrode was reduced appropriately to seal the cells; the electrode capacitance was compensated, and then the negative pressure inside the microelectrode was increased to break the cell membrane; and a whole-cell recording mode was formed. The membrane potential was held at 0 mV using a patch clamp amplifier, and the membrane potential was measured between −100 mV and +100 mV by pulsing the software in order to create a current–voltage (I–V) relationship. Cellular current assays were performed by compensating for whole-cell membrane capacitance and series resistance, correcting for leakage current by adding the chloride channel activators trichothecene and ionomycin to the cell bath and comparing the current to that of a typical chloride ion to detect its current characteristics. Tea residue filtrate was added to the cell bath solution and whole-cell recordings were made at room temperature using a membrane clamp amplifier on cells that emitted EGFP fluorescence.

### 2.15. Statistical Analysis

Data are expressed as means, and SEM is the total standard error of the total sample mean. Data were analyzed using SPSS 25.0 software (IBM Corp., Chicago, IL, USA) to test the data according to normal distribution, then analyzed using an independent-samples *t*-test to determine statistical differences between groups according to Duncan’s method, where 0.05 ≤ *p* < 0.10 is a trend of difference, *p* < 0.05 is a significant difference, and *p* < 0.01 is a highly significant difference.

## 3. Results

### 3.1. Effect of Growth Performance

Table 4 shows the effect of feed supplementation with tea residue on growth performance. Supplementation of the diet with 1 kg/t of tea residue had no significant effect on body weight (BW), average daily gain (ADG), average daily feed intake (ADFI) and feed conversion ratio (G:F) of weaned piglets (*p* > 0.05).

### 3.2. Diarrhea Rate

Compared to the basal diet group, the addition of 1 kg/t tea residue to the diet significantly reduced the diarrhea rate in weaned piglets at d 1–7 and d 1–28 (*p* < 0.01); however, there was no significant effect on the diarrhea rate at d 8–28 (Table 5).

### 3.3. Nutrient Digestibility

The effect of tea residue on the nutrient digestibility of weaned piglets is shown in Table 6. There were no significant differences in crude protein, crude fat, crude fiber and total energy digestibility between the two groups (*p* > 0.05).

### 3.4. Plasma Biochemical Indexes

As shown in Table 7, the addition of tea residue to the diet had no significant effect on the plasma biochemical indexes of total protein, albumin, globulin, cholesterol, triglycerides, AST, ALT, ALP, HDL and LDL in weaned piglets (*p* > 0.05).

### 3.5. Effect on Plasma Antioxidant Indexes

As shown in Table 8, compared with the basal diet group, there was no significant effect (*p* > 0.05) on antioxidant indexes such as CAT, GSH-Px, T-SOD, T-AOC and MDA in weaned piglets fed the tea residue diet, but the level of glutathione (GSH) was significantly reduced at d 28 (*p* < 0.05).

### 3.6. Effect on Plasma Immune Indexes

As shown in Table 9, the addition of tea residue to the diet had no significant effect (*p >* 0.05) on IgA, IgG and IgM plasma immune indexes in weaned piglets.

### 3.7. Effect on the Morphological Structure of the Intestine

As shown in Table 10, the addition of 1 kg/t of tea residue to the diet fed to piglets had no significant effect on the jejunum, ileum villus height, crypt depth and VH/CD (*p* > 0.05).

### 3.8. Effect on Fecal Mineral Fraction

As shown in Table 11, the addition of tea residue to the diet reduced the fecal chloride content of weaned piglets very significantly (*p* < 0.01) compared to the basal diet group, but there was no significant difference in the content of mineral fractions such as calcium, phosphorus and sodium in the feces (*p* > 0.05).

### 3.9. Effect on mRNA Expression of Colonic Chloride Channels

**Figure 1:**
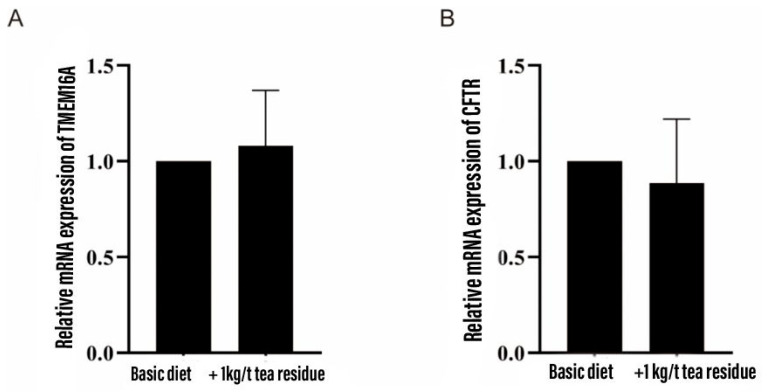
Effect of tea residue on the expression of chloride channel mRNA in colon of weaned piglets. (**A**) *TMEM16A* and (**B**) *CFTR*.

As shown in Figure 1, the addition of tea residue to the diet had no significant effect on the mRNA expression of the colonic chloride channels *TMEM16A* and *CFTR* in weaned piglets.

### 3.10. Effect on Electrophysiological Activity of Colonic Chloride Channels

**Figure 2:**
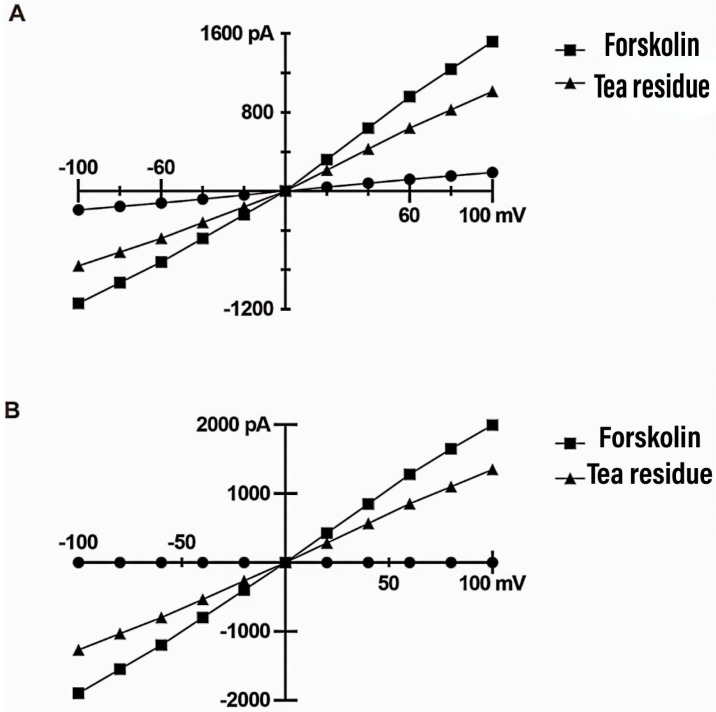
HT-29 cell voltage–current (I–V) curve. (**A**). TMEM16A whole-cell membrane clamp for recording assay results. (**B**). CFTR whole-cell membrane clamp for recording assay results.

Figure 2 shows the current–voltage (I–V) curves obtained for *TMEM16A* and *CFTR* under different treatment conditions with a cell clamp potential of 0 mV and a test potential obtained by gradually changing from −100 mV to +100 mV (step voltage of 20 mV). Whole-cell membrane clamp recordings showed that the *TMEM16A* ion channel current was positively correlated with the change in voltage, with a small increase at negative charges and an increase in current change with increasing positive voltage. Ionomycin activated *TMEM16A* to produce a chloride current, and this current was significantly reduced after tea residue filtrate treatment, indicating that *TMEM16A* could be significantly inhibited by tea residue. Whole-cell membrane clamp recordings of *CFTR* ion channels showed that the current was positively correlated with the change in voltage. Forskolin activated *CFTR* channels to generate a chloride current, and this current was significantly reduced after tea residue filtrate treatment, indicating that *CFTR* can be significantly inhibited by tea residue.

## 4. Discussion

Weaning is an important stage of piglet growth. The gastrointestinal tract is the largest immune organ of the organism and protects the body from various pathogens, antigens, endotoxins and potentially harmful microorganisms [26]. The main target of weaning stress is in the intestine, which causes some damage to the morphological structure of the piglet intestine and affects the intestinal function [22], manifested by oxidative damage, barrier destruction and reduced digestion and absorption capacity [27]. This results in reduced feed intake, slower growth and increased morbidity in piglets. Under stressful conditions, piglets are more likely to be infected with pathogenic bacteria that further damage the intestinal structure and function, resulting in or aggravating vomiting and diarrhea, which can endanger piglet health, increase piglet mortality and cause huge economic losses [3,28]. In addition, these factors also have a critical impact on the growth performance of piglets [29]. Feeding antibiotics can effectively relieve post-weaning diarrhea and improve piglet growth performance, but the long-term overuse of antibiotics leads to bacterial resistance and antibiotic residues in animal products, threatening human health. Some studies have shown that fermented tea, which has various functional properties such as anti-inflammatory potential and antioxidant activity [30], may contribute to the improvement of intestinal function.

Tea is an important cash crop in China with the advantages of being non-toxic and green. Tea polyphenols have functions such as antioxidant and antibacterial. However, polyphenolic substances can also affect the taste of tea, and when tea polyphenols are present in excess, they can exhibit a bitter taste, and when tea residue is added to the ration, it can affect its palatability. Feeding diets with added tea residue can have different degrees of effects on different kinds of animals. Studies have shown that fermented herbal tea residue can effectively reduce the respiratory rate and rectal temperature of fattening cattle under heat stress and increase daily feed intake and daily weight gain [31]. When black or green tea and their tea residues were added to sheep diets, their rumen function was optimized and methane emissions were reduced [32]. As explored by this experiment, there was no significant difference in the growth performance of piglets fed diets supplemented with tea residue compared to the control group, which is consistent with Zhaoming Yan [33], indicating that tea residue has no adverse effect on the growth performance of weaned piglets.

Digestibility is an important index for measuring the digestibility of animals and feed digestibility. When piglets are weaned, because their digestive system is not yet mature, piglets often have problems such as insufficient secretion of gastric acid and low activity of digestive enzymes in the intestine, which cause a decrease in the digestion and absorption of nutrients in the diet of weaned piglets and affect their growth performance [34]. Mohammed Rashed Chowdhury showed that feeding a diet supplemented with heat-treated green tea increased the apparent crude protein digestibility of Black Bengal goats [35]. Studies showed that the addition of fermented tea residue (equal percentage replacement of concentrate supplement) had no significant effect on the digestibility of organic matter, crude protein and dry matter in lake goats [36]. This was similar to the results of the present study, where tea residue did not cause significant differences in crude protein, crude fat, crude fiber and total energy digestibility in weaned piglets.

Plasma biochemical indicators reflect the health status of an organism. The addition of tea residue to the diet had no significant effect on the plasma biochemical indexes of total protein, albumin, globulin, cholesterol, triglycerides, AST, ALT, ALP, HDL and LDL in weaned piglets. This is basically consistent with the results of Wang Guiyun’s study, thus indicating that tea residue has no negative effect on the plasma biochemical functions of weaned piglets.

The antioxidant capacity of growing pigs is fundamental for maintaining the normal metabolic state to protect a pig’s health [37]. The antioxidant defense system in the body has a key role in the normal growth of animals and the removal of harmful substances such as oxygen free radicals from the body. Tea polyphenols can improve antioxidant capacity and glutathione peroxidase activity and reduce malondialdehyde content in growing fattening pigs, and their antioxidant capacity is influenced by the spatial configuration, which is generally positively correlated with the number of hydroxyl groups [38,39]. When fermented tea residue was added to the diets of fattening pigs, it significantly increased glutathione peroxidase activity and total antioxidant capacity [40]. In the present study, the addition of tea residue had no significant effect on the activities of various antioxidant enzymes in weaned piglets compared with the control group, but it increased the plasma glutathione content at day 28, indicating that tea residue has positive effects on antioxidant activity and maintaining normal physiological functions of the body.

The effect of tea and tea residue is mainly dependent on the active ingredients it contains, such as tea polysaccharides, tea polyphenols, L-theanine, tea pigments and other common components. When Fuzhuan tea polysaccharide was added to the diet of cyclophosphamide-treated mice, it restored the levels of cytokines and immunoglobulins and effectively regulated the immune function of mice [41]. In the present study, however, there was no significant effect on plasma immune indexes in weaned piglets.

The intestinal barrier is an important way for the intestinal epithelium to effectively prevent the invasion of toxic and harmful substances [42]. A normal intestinal structure is an important foundation for the healthy and fast growth of piglets, and weaning stress in piglets can damage the intestinal structure with problems such as intestinal villus atrophy and increased crypt depth, which can have an impact on digestion, absorption, secretion and barrier function [43]. Yixuan Bai found that Fuzhuan tea polysaccharides improved cyclophosphamide-induced intestinal damage in mice and effectively restored their intestinal morphology [41]. Green tea polyphenols dose-dependently improved intestinal epithelial homeostasis in mice and showed superior anti-inflammatory effects and colonic barrier integrity [44]. In the present study, no differential changes in the intestinal morphology of piglets fed tea residue were observed compared to the control group. Thus, it appears that tea residue has no adverse effect on the morphological structure of the intestinal mucosa of piglets.

The maintenance of the normal state of the intestinal epithelium is important to the activities of key physiological processes, such as digestion, absorption and immune responses [45]. Much evidence suggests that herbs such as tea can be effective against diarrhea [19]. Considering the special method of making Fuzhuan tea, it is also possible that it is the result of the action of fermenting microorganisms and their metabolites and active substances. There are studies to reduce diarrhea in the first two weeks after weaning by developing feeding strategies [46]. In the present study, by recording diarrhea in piglets, it was found that the addition of tea residue to the diet significantly reduced the diarrhea rate from 1 to 7 d and 1 to 28 d. This shows that tea residue has a good anti-diarrheal effect and has a wide scope for feed development and the development of anti-diarrheal products. In this study, the addition of tea residue to the diet also significantly reduced the fecal chloride content in weaned piglets. From this, we can tentatively determine that tea residue can promote the absorption of chloride ions, and since watery diarrhea is mainly due to the excessive excretion of sodium and chloride ions from the intestine, it is tentatively inferred that it is caused by abnormal expression or dysfunction of chloride channels in the colon.

*TMEM16A* is a calcium-dependent chloride channel whose activity is controlled by cytoplasmic calcium concentration. It plays an important physiological role in many organ systems. Overexpression of *TMEM16A* will lead to pulmonary hypertension [47], hypertension [48], cancer [49] and so on. *TMEM16A*-mediated CaCC is regulated by calmodulin-dependent protein kinase II (CaMKII) and protein phosphatase 1/protein phosphatase 2A (PP1/PP2A) [50]. *CFTR* can be regulated by AMP protein-activated kinase (AMPK) [51]; it is the main channel for chloride secretion in the intestine. It was found that the addition of tea residue to the diet significantly reduced the fecal chlorine content in weaned piglets. From this, we can tentatively determine that tea residue can promote the absorption of chloride ions, and since watery diarrhea is mainly due to the excessive excretion of sodium and chloride ions from the intestine, it is tentatively inferred that it is due to the abnormal expression or dysfunction of chloride channels in the colon. However, there was no significant effect on the relative expression of mRNA of the colonic ion channels *TMEM16A* and *CFTR* in weaned piglets, and combined with cell culture and electrophysiological experiments, it was further verified that tea residue extract could significantly inhibit the opening of *TMEM16A* and *CFTR* ion channels in HT-29 cells, thus improving diarrhea in weaned piglets.

## 5. Conclusions

In conclusion, the addition of tea residue to the diet had no effect on the growth performance and digestibility of piglets, but to some extent, it could enhance the antioxidant function of piglets and significantly reduce the rate of post-weaning diarrhea. Dietary tea residue could significantly reduce the content of chloride ions in feces but had no significant effect on the relative mRNA expression of the colon ion channels *TMEM16A* and *CFTR* in weaned piglets. Combined with cell culture and electrophysiological experiments, it was further verified that tea residue extract could significantly inhibit the opening of the *TMEM16A* and *CFTR* ion channels in HT-29 cells, thus improving diarrhea in weaned piglets.

## Figures and Tables

**Table 1:** Ingredients and nutrient composition of experimental piglets.

Projects	Content
Component, %	
Corn	61.39
Whey powder	5.00
Puffed soybeans	5.00
Soybean meal	15.33
Fish meal	3.00
Plasma protein powder	3.00
Calcium hydrogen phosphate	1.70
Zeolite powder	1.00
Stone powder	1.00
Active yeast	2.28
Lysine hydrochloride	0.23
DL-Methionine	0.07
Compound premixes ^1^	1.00
Total	100
Nutritional level	
Crude protein, %	20.00
Digestive energy, Mcal/kg	3.25
Lysine ^2^, %	1.30
Methionine ^2^ + cysteine ^2^, %	0.75
Tryptophan ^2^, %	0.29
Threonine ^2^, %	0.85
Ca, %	1.00
Total phosphorus ^2^, %	0.75
Na, %	0.27

^1^ per kg of basic diet, 10,000 IU vitamin A, 80 IU vitamin E, 1000 IU vitamin D_3_, 25 mg pantothenic acid, 2.0 mg vitamin K_3_, 12 mg riboflavin, 0.03 mg vitamin B_12_, 0.25 mg biotin, 1.6 mg folic acid, 2.25 mg vitamin B_6_, 40 mg niacin, 3.0 mg thiamine, 300 mg choline chloride, 150 mg FeSO_4_, 100 mg ZnSO_4_, 30 mg MnSO_4_, 0.5 mg KIO_3_, 0.3 CoSO_4_, 25 mg CuSO_4_, 0.3 mg Na_2 _SeO3 and 4.0 mg ethoxyquinoline were added. ^2^ Standard ileal digestibility of amino acids.

**Table 2:** Primers used for real-time PCR analysis.

Genes	Primers	Sequence, 5′-3′	Size, bp
*β-actin*	Forward	AGTTGAAGGTGGTCTCGTGG	216
	Reverse	TGCGGGACATCAAGGAGAAG	
*TMEM16A*	Forward	CAAAACCCGGAGCACAATCG	149
	Reverse	CGTGCTCCCCTTCGTAGTC	
*CFTR*	Forward	CTACGCTGGTTCCAAATGCG	244
	Reverse	TAGGTTGATCTCCTTCTGCCG	

**Table 3:** PCR primer design.

Genes	Primers	Sequence 5′-3′
*TMEM16A*	Forward	ATTGCTAGCGCCACCATGGAGGAGACGACGCTGGA
	Reverse	AATTATAGGATCCGCAGGGGGCCCCCGGAA
*CFTR*	Forward	CCGCTCGAGGCCACCATGTTCTATGGAATCATATTATAT
	Reverse	TAGACCGGTCAAGTCTTGTTTCTTGCA

**Table 4:** Effect of tea residue on growth performance of weaned piglets.

Projects	Basic Diet	+1 kg/t Tea Residue	*p* Value
BW, kg			
d 1	6.52 ± 0.12	6.56 ± 0.14	0.396
d 7	6.77 ± 0.14	6.75 ± 0.17	0.783
d 28	10.25 ± 0.24	10.21 ± 0.36	0.734
ADG, g/d			
1~7 d	13.82 ± 2.89	13.46 ± 2.61	0.699
8~28 d	173.13 ± 6.85	170.79 ± 6.70	0.309
1~28 d	133.13 ± 8.57	130.56 ± 14.47	0.520
ADFI, g/d			
1~7 d	78.38 ± 3.66	78.52 ± 3.73	0.911
8~28 d	420.09 ± 11.69	415.85 ± 10.26	0.256
1~28 d	381.14 ± 7.56	384.30 ± 7.87	0.227
G:F, g/g			
1~7 d	5.91 ± 1.24	6.03 ± 1.14	0.765
8~28 d	2.43 ± 0.12	2.44 ± 0.13	0.832
1~28 d	2.87 ± 0.18	2.98 ± 0.35	0.265

BW = body weight; ADG = average daily gain; ADFI = average daily feed intake; G:F = feed intake-to-body gain ratio.

**Table 5:** Effect of tea residue on diarrhea rate of weaned piglets.

Projects	Basic Diet	+1 kg/t Tea Residue	*p* Value
1–7 d	32.34 ± 4.16	26.35 ± 2.08	<0.001
8–28 d	14.70 ± 1.12	15.55 ± 2.19	0.151
1–28 d	19.94 ± 0.60	17.98 ± 2.42	0.002

Diarrhea rate = number of piglets with diarrhea during the trial period/(total number of pigs × number of trial days) × 100%.

**Table 6:** Effect of tea residue on nutrient digestibility of weaned piglets.

Projects	Basic Diet	+1 kg/t Tea Residue	*p* Value
Crude protein			
d 7	37.88 ± 3.67	36.90 ± 3.41	0.413
d 28	73.63 ± 3.83	72.31 ± 2.71	0.240
Crude fat			
d 7	57.91 ± 2.47	58.62 ± 1.80	0.333
d 28	59.53 ± 2.52	60.53 ± 2.17	0.209
Crude fiber			
d 7	17.14 ± 3.00	16.54 ± 2.30	0.500
d 28	38.06 ± 2.51	36.95 ± 3.13	0.248
Total energy			
d 7	59.12 ± 2.67	59.61 ± 2.46	0.570
d 28	84.20 ± 2.13	83.81 ± 2.05	0.579

**Table 7:** Effect of dietary tea residue on plasma biochemical parameters of weaned piglets.

Projects	Basic Diet	+1 kg/t Tea Residue	*p* Value
TP, g/L			
d 7	49.88 ± 0.59	49.94 ± 0.67	0.763
d 28	45.29 ± 2.98	43.96 ± 2.57	0.159
ALB, g/L			
d 7	28.65 ± 0.37	28.80 ± 0.38	0.244
d 28	23.69 ± 0.36	23.81 ± 0.33	0.304
GLO, g/L			
d 7	21.56 ± 0.49	21.35 ± 0.57	0.245
d 28	21.44 ± 0.61	21.60 ± 0.68	0.473
TC, mmol/L			
d 7	1.59 ± 0.03	1.60 ± 0.03	0.229
d 28	1.77 ± 0.09	1.79 ± 0.09	0.491
TG, mmol/L			
d 7	0.40 ± 0.03	0.41 ± 0.03	0.090
d 28	0.38 ± 0.02	0.38 ± 0.02	0.568
AST, U/L			
d 7	94.47 ± 11.46	98.62 ± 12.20	0.301
d 28	105.21 ± 5.63	105.88 ± 6.03	0.735
ALT, U/L			
d 7	45.98 ± 2.73	45.31 ± 3.13	0.495
d 28	39.55 ± 3.32	38.82 ± 2.94	0.488
ALP, U/L			
d 7	255.65 ± 12.06	258.73 ± 12.76	0.461
d 28	181.41 ± 19.98	182.90 ± 19.14	0.821
HDL, mmol/L			
d 7	0.64 ± 0.02	0.64 ± 0.02	0.665
d 28	0.40 ± 0.03	0.39 ± 0.03	0.403
LDL, mmol/L			
d 7	0.66 ± 0.01	0.66 ± 0.01	0.815
d 28	0.97 ± 0.06	0.96 ± 0.06	0.751

TP = total protein; ALB = albumin; GLO = globulin; TC = cholesterol; TG = triglyceride; AST = glutathione aminotransferase; ALT = alanine aminotransferase; ALP = alkaline phosphatase; HDL = high-density lipoprotein; LDL = low-density lipoprotein.

**Table 8:** Effect of tea residue on plasma antioxidant indexes of weaned piglets.

Projects	Basic Diet	+1 kg/t Tea Residue	*p* Value
CAT, U/mL			
d 7	51.93 ± 5.41	53.08 ± 4.95	0.510
d 28	50.38 ± 7.60	51.62 ± 7.54	0.626
GSH, μmol/L			
d 7	4.26 ± 1.58	3.59 ± 1.39	0.185
d 28	2.52 ± 0.28	2.73 ± 0.19	0.012
GSH-Px, U/mL			
d 7	356.07 ± 16.43	352.12 ± 16.91	0.482
d 28	443.83 ± 46.94	444.10 ± 52.12	0.987
T-SOD, U/mL			
d 7	116.80 ± 0.13	116.84 ± 0.20	0.505
d 28	116.81 ± 0.17	116.77 ± 0.17	0.552
T-AOC, U/mL			
d 7	5.41 ± 1.96	5.25 ± 1.97	0.806
d 28	4.84 ± 0.80	4.48 ± 0.73	0.168
MDA, nmol/mL			
d 7	46.86 ± 3.71	47.38 ± 3.10	0.651
d 28	48.08 ± 3.99	49.23 ± 4.58	0.431

**Table 9:** Effect of tea residue on plasma immune index of weaned piglets.

Projects	Basic Diet	+1 kg/t Tea Residue	*p* Value
IgA, mg/mL			
d 7	0.75 ± 0.05	0.73 ± 0.06	0.252
d 28	1.93 ± 0.19	1.95 ± 0.20	0.737
IgG, mg/mL			
d 7	3.95 ± 0.21	3.89 ± 0.25	0.394
d 28	2.20 ± 0.16	2.22 ± 0.18	0.747
IgM, mg/mL			
d 7	0.58 ± 0.06	0.56 ± 0.07	0.317
d 28	0.76 ± 0.06	0.71 ± 0.06	0.849

IgA = immunoglobulin A; IgG = immunoglobulin G; IgM = immunoglobulin M.

**Table 10:** Effect of tea residue on intestinal morphology of weaned piglets.

Projects	Basic Diet	+1 kg/t Tea Residue	*p* Value
jejunum			
villus height, μm	329.57 ± 12.49	334.32 ± 13.51	0.282
crypt depth, μm	330.09 ± 9.86	334.07 ± 14.56	0.345
VH/CD	1.00 ± 0.05	1.00 ± 0.06	0.849
ileum			
villus height, μm	262.25 ± 7.85	260.04 ± 7.82	0.404
crypt depth, μm	285.41 ± 6.46	287.06 ± 6.31	0.444
VH/CD	0.92 ± 0.04	0.91 ± 0.03	0.247

VH/CD = villus height-to-crypt depth ratio.

**Table 11:** Effect of tea residue on mineral composition of feces of weaned piglets.

Projects	Basic Diet	+1 kg/t Tea Residue	*p* Value
Ca, mg/kg	5072.89 ± 565.29	4979.61 ± 600.21	0.634
P, mg/kg	4088.00 ± 260.73	4111.94 ± 207.51	0.762
Na, mg/kg	4056.06 ± 220.76	4112.72 ± 233.29	0.459
Cl, mg/kg	4051.67 ± 255.08	3205.25 ± 153.20	<0.001

## Data Availability

Data are contained within the article.
